# Inhibition of SARS-CoV-2 Nsp9 ssDNA-Binding Activity and Cytotoxic Effects on H838, H1975, and A549 Human Non-Small Cell Lung Cancer Cells: Exploring the Potential of *Nepenthes miranda* Leaf Extract for Pulmonary Disease Treatment

**DOI:** 10.3390/ijms25116120

**Published:** 2024-06-01

**Authors:** Hsin-Hui Su, En-Shyh Lin, Yen-Hua Huang, Yi Lien, Cheng-Yang Huang

**Affiliations:** 1 Department of Pharmacy, Chia Nan University of Pharmacy and Science, Tainan City 717, Taiwan; 2 Department of Beauty Science, National Taichung University of Science and Technology, Taichung City 403, Taiwan; 3 Department of Biomedical Sciences, Chung Shan Medical University, Taichung City 402, Taiwan; 4 Department of Biological Sciences, Purdue University, West Lafayette, IN 47907, USA; 5 Department of Medical Research, Chung Shan Medical University Hospital, Taichung City 402, Taiwan

**Keywords:** *Nepenthes*, anticancer, SARS-CoV-2, Nsp9, NSCLC, AntoDock, lupenone, stigmast-5-en-3-ol, plumbagin

## Abstract

Carnivorous pitcher plants from the genus *Nepenthes* are renowned for their ethnobotanical uses. This research explores the therapeutic potential of *Nepenthes miranda* leaf extract against nonstructural protein 9 (Nsp9) of SARS-CoV-2 and in treating human non-small cell lung carcinoma (NSCLC) cell lines. Nsp9, essential for SARS-CoV-2 RNA replication, was expressed and purified, and its interaction with ssDNA was assessed. Initial tests with myricetin and oridonin, known for targeting ssDNA-binding proteins and Nsp9, respectively, did not inhibit the ssDNA-binding activity of Nsp9. Subsequent screenings of various *N. miranda* extracts identified those using acetone, methanol, and ethanol as particularly effective in disrupting Nsp9’s ssDNA-binding activity, as evidenced by electrophoretic mobility shift assays. Molecular docking studies highlighted stigmast-5-en-3-ol and lupenone, major components in the leaf extract of *N. miranda*, as potential inhibitors. The cytotoxic properties of *N. miranda* leaf extract were examined across NSCLC lines H1975, A549, and H838, focusing on cell survival, apoptosis, and migration. Results showed a dose-dependent cytotoxic effect in the following order: H1975 > A549 > H838 cells, indicating specificity. Enhanced anticancer effects were observed when the extract was combined with afatinib, suggesting synergistic interactions. Flow cytometry indicated that *N. miranda* leaf extract could induce G2 cell cycle arrest in H1975 cells, potentially inhibiting cancer cell proliferation. Gas chromatography–mass spectrometry (GC–MS) enabled the tentative identification of the 19 most abundant compounds in the leaf extract of *N. miranda*. These outcomes underscore the dual utility of *N. miranda* leaf extract in potentially managing SARS-CoV-2 infection through Nsp9 inhibition and offering anticancer benefits against lung carcinoma. These results significantly broaden the potential medical applications of *N. miranda* leaf extract, suggesting its use not only in traditional remedies but also as a prospective treatment for pulmonary diseases. Overall, our findings position the leaf extract of *N. miranda* as a promising source of natural compounds for anticancer therapeutics and antiviral therapies, warranting further investigation into its molecular mechanisms and potential clinical applications.

## 1. Introduction

Carnivorous plants like *Nepenthes*, rich in secondary metabolites, perform multiple functions, including attracting, capturing, and digesting prey, defending against pathogens and herbivores, and luring pollinators [1,2]. Known for their diverse biological activities, these compounds offer medicinal potential [1]. Traditionally, *Nepenthes* plants have been used in folk medicine to treat ailments such as stomachaches and fevers [3,4]. Due to their longstanding use in traditional medicine, extracts from *Nepenthes* are considered relatively safe for pharmaceutical applications, likely presenting minimal side effects for humans [4]. Recent extensive research into the biological effects of both whole extracts and individual compounds from these plants underscores the need for further exploration of their therapeutic potential [5,6,7]. Accordingly, this study aims to explore the potential benefits of the extract of *Nepenthes miranda*, a novel cultivar [8] from a hybrid of *N. maxima* and *N. northiana*, in treating pulmonary diseases by targeting the severe acute respiratory syndrome coronavirus 2 (SARS-CoV-2) Nsp9 protein and assessing its cytotoxic activity against lung carcinoma cells.

SARS-CoV-2 [9], the virus responsible for COVID-19, is a highly contagious and pathogenic coronavirus that continues to impact the world with ongoing pandemics and over 6.9 million deaths to date. The development of mRNA vaccines, Comirnaty (BNT162b2) and Spikevax (mRNA-1273), which train the immune system to recognize and combat COVID-19 by mimicking the virus’s spike protein, has contributed to a reduction in mortality rates [10]. Despite this progress, the virus’s ability to mutate has led to variants like Omicron, including subvariants EG.5 and JN.1 [11,12], that can evade vaccine-induced immunity, highlighting the need for ongoing therapeutic development [13,14]. The SARS-CoV-2 genome, encoding 29 proteins, presents multiple targets for drug discovery, including its nonstructural proteins (Nsp) essential for viral replication and interaction with host cells [15]. Notably, nirmatrelvir, an inhibitor of the virus’s main protease (Nsp5) included in Paxlovid by Pfizer, has received approval for treating high-risk COVID-19 patients [16]. However, the development of resistance to Paxlovid [17,18] highlights the critical need for new antiviral agents and the pursuit of complementary and alternative medicines to combat the evolving threat of SARS-CoV-2 [13,14,15,19,20]. This underscores the importance of exploring new inhibitors and alternative medicines targeting viral proteins.

Nsp9, a conserved coronaviral replicase essential for priming viral RNA synthesis, functions as a critical component of the multi-subunit viral replication/transcription complex [21,22,23,24,25]. This makes it a promising new target for developing treatments that could potentially suppress SARS-CoV-2 infection. Despite its potential, there are currently no approved drugs targeting Nsp9. Known as an RNA-binding protein, Nsp9 is also covalently linked to the 5’ ends of both positive- and negative-sense RNAs generated during infection [22]. It has demonstrated the capability to bind single-stranded DNA (ssDNA) as well [26]. With the availability of Nsp9’s crystal structure, the prospects for developing effective therapeutics are enhanced. Notably, the natural compound oridonin has been shown to bind to Nsp9, inhibiting its function as a substrate for the Nidovirus RdRp-Associated Nucleotidyltransferase (NiRAN) domain of Nsp12 [27]. Ongoing efforts to discover more inhibitors are crucial.

The crystal structure of Nsp9 reveals a single beta-barrel fold that superficially resembles the oligonucleotide/oligosaccharide-binding (OB) fold [26]. This structure [28] is common among ssDNA-binding proteins (SSBs) that bind to ssDNA [29,30,31,32,33,34,35,36]. Given the structural resemblance between SSB and Nsp9, we sought to explore whether inhibitors of SSB could also suppress the nucleic acid binding activity of Nsp9. Both natural compounds and crude plant extracts, such as myricetin [29] and the extract of *N. miranda* [37], have been effective as SSB inhibitors. Investigating whether these SSB inhibitors can also act as novel Nsp9 inhibitors is a promising avenue for research.

According to the World Health Organization (WHO), lung cancer is the leading cause of cancer death globally, responsible for 1.8 million deaths annually [38]. Non-small cell lung cancer (NSCLC), which accounts for approximately 85% of lung cancer cases, is typically managed with surgery, chemotherapy, radiation, and targeted therapy [39,40]. Modern oncology heavily depends on effective anticancer drugs; however, NSCLCs often exhibit limited responsiveness to chemotherapy [41,42]. Single-agent therapies can prompt cancer cells to activate alternative survival pathways, leading to drug resistance [43]. Approximately 90% of cancer therapy failures and 80–90% of cancer-related deaths are attributed to this resistance [44]. Thus, integrating multiple chemotherapeutic agents should be considered for treatment. Recently, natural compounds have gained attention as potential anti-lung cancer agents and alternative therapeutic options [45,46,47,48]. Natural extracts are advantageous in targeting multiple pathways in cancer cells [49]. Clinical trials have suggested that combinations of chemotherapeutic drugs with natural products may achieve effects similar to those of conventional chemotherapy but with fewer side effects [44,50]. Natural products, therefore, provide an alternative approach, with unique opportunities to discover compounds with drug-like properties [51]. Significantly, more than 30% of all Food and Drug Administration (FDA)-approved drugs are either natural products or their derivatives [52,53,54]. These phytochemicals might also address challenges such as drug resistance, a prevalent issue in lung cancer treatment. Accordingly, this study examined the cytotoxic effects of *N. miranda* extract both alone and in combination with the clinical anti-lung cancer drug afatinib, to assess any synergistic effects that may be beneficial in combating NSCLC.

This study, identifying Nsp9 inhibition, cytotoxic activity against lung cancer cells, and the synergistic effect with afatinib, extends the potential applications of *N. miranda* leaf extract beyond its traditional folk medicine uses to potentially treat pulmonary diseases.

## 2. Results

### 2.1. Cloning, Expression, and Purification of the Recombinant Nsp9 Protein from SARS-CoV-2

**Figure 1:**
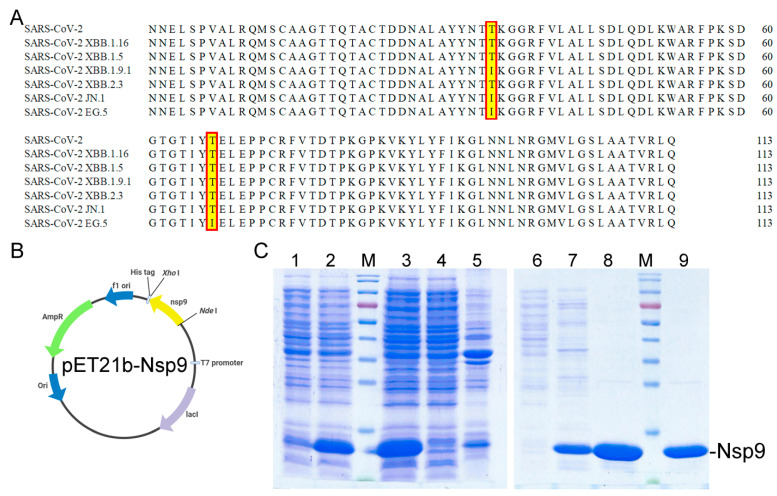
Recombinant Nsp9 Protein from SARS-CoV-2. (**A**) Amino acid sequence alignment of Nsp9 from SARS-CoV-2. Sequence alignments across variants demonstrate high conservation with minor mutations (T35I and T67I) observed. (**B**) Construction of the expression plasmid pET21b-Nsp9. The synthesized gene, including NdeI and XhoI restriction sites, was ligated into the pET21b expression vector. (**C**) Protein expression and purification. Analysis was performed using 12% SDS-PAGE. The analysis includes bacterial culture before (lane 1) and after IPTG induction (lane 2), sonicated and centrifuged *E. coli* cells yielding supernatant (lane 3), supernatant applied to Ni-NTA column with analysis of flowthrough (lane 4), and pellet content (lane 5). Chromatographic steps with serial elutions in 5 mM imidazole buffer (20 mM Tris–HCl, 5 mM imidazole, and 0.5 M NaCl, pH 7.9; lane 6), 60 mM (lane 7), 100 mM (lane 8), and 200 mM (lane 9) imidazole were performed to obtain purified Nsp9. Elutions with 100 mM and 200 mM imidazole were effective in achieving homogeneity.

The SARS-CoV-2 virus, composed of 29 proteins including 16 nonstructural, 4 structural, and 9 accessory proteins [55], harbors numerous potential therapeutic targets due to their vital roles in viral replication and host cell machinery manipulation [15]. The virus’s nonstructural proteins (Nsps), sharing a 96% nucleotide sequence identity with SARS-like bat coronaviruses, present druggable targets for drug development [56]. This study focuses on exploiting the *N. miranda* extract to inhibit Nsp9, a key RNA-binding protein essential for viral replication [22]. Initial sequence analysis (Figure 1A) was conducted to assess the conservation of Nsp9 since its emergence in 2019 (gene ID 43740578 and the nucleotides 12421–12759), as referenced in the National Center for Biotechnology Information (NCBI). The anticipated monomeric form of Nsp9 consists of 113 amino acids, weighing approximately 12.6 kDa. The emergence of SARS-CoV-2 variants, such as Omicron with EG.5 [57] and JN.1 [58,59] subvariants, challenges the efficacy of existing vaccines and antibodies due to mutations enabling vaccine escape. However, sequence alignments of Nsp9 across these variants show a high degree of conservation, with only minor mutations (T35I in JN.1 and T35I, T67I in EG.5), suggesting a stable sequence amidst evolutionary pressures and vaccine development (Figure 1A). To proceed with inhibition studies, the Nsp9 gene was synthesized, cloned into a pET21b vector (pET21b-Nsp9), and expressed in *Escherichia coli*, resulting in a protein tagged with eight additional residues (LEHHHHHH) at the C-terminus for purification purposes (Figure 1B), yielding a final protein size of 13.6 kDa. Following overexpression in *E. coli*, Nsp9 was purified through a single-step Ni^2+^-affinity chromatography (Figure 1C) and dialyzed into a storage buffer for subsequent experiments, resulting in more than 30 mg of purified protein per liter of bacterial culture.

### 2.2. ssDNA-Binding Activity of Nsp9 and Inhibition Tests Using Myricetin and Oridonin

**Figure 2:**
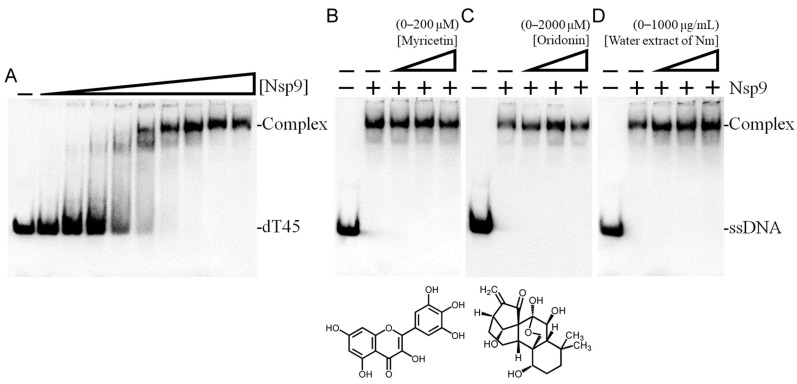
ssDNA binding activity of Nsp9. (**A**) Purified recombinant Nsp9 at various concentrations (0, 3, 6, 13, 25, 50, 75, 100, 150, 200 μM) was incubated with biotin-labeled ssDNA dT45 at 37 °C for 60 min. An increase in Nsp9 concentration resulted in a notable band shift, indicative of ssDNA binding. Binding constants ([Protein]_50_) were determined through linear interpolation based on the protein concentration. Binding assays were also conducted with the inclusion of (**B**) myricetin, (**C**) oridonin, or (**D**) water extract from *N. miranda* leaves in the protein solution, using 100 μM Nsp9. Myricetin and oridonin were dissolved in 10% dimethyl sulfoxide (DMSO). Nm denotes *N. miranda*.

Electrophoretic mobility shift analysis (EMSA) was employed to assess the binding affinity of purified Nsp9 for nucleic acids across a range of concentrations (0–200 μM). Recognized for its ability to bind ssRNA, Nsp9 is similarly adept at binding ssDNA [26]. To circumvent potential RNA degradation and unintended secondary structure formation during electrophoresis, a ssDNA homopolymer dT45 was utilized for the assay instead of ssRNA. The EMSA method hinges on the premise that stable protein–DNA complexes exhibit slower electrophoretic movement than free DNA. A progressive increase in Nsp9 concentration demonstrated a significant band shift, signaling stable complexation with the ssDNA. This shift facilitated the calculation of Nsp9’s ssDNA-binding affinity ([Protein]_50_) to dT45, which was established at 33.5 ± 2.5 μM (Figure 2A). For inhibition experiments, Nsp9 was used at a 100 μM concentration, proven to effectively bind ssDNA dT45. The flavonol myricetin, known to inhibit SSB (a ssDNA-binding protein sharing structural similarities with Nsp9) at an IC_50_ of 2.8 μM [29,36], was initially tested for its inhibitory effect on Nsp9 (Figure 2B). Despite its efficacy against SSB, myricetin failed to inhibit Nsp9, even at 200 μM, suggesting it does not act as an Nsp9 inhibitor at these concentrations. Oridonin, identified as a potent Nsp9 ligand with a *K*_d_ of 7.2 ± 1.0 μM [27], was also tested for its ability to inhibit Nsp9’s ssDNA-binding activity at varying concentrations (0–400 μM). Nevertheless, oridonin showed no impact on Nsp9’s ssDNA-binding capability (Figure 2C), indicating it too does not inhibit Nsp9. Therefore, neither myricetin (an SSB inhibitor) nor oridonin (an Nsp9 binder) inhibited Nsp9’s ssDNA-binding activity, underscoring the need for ongoing exploration of effective inhibitors (Table 1).

### 2.3. Exploring Nsp9 Inhibition with N. miranda Plant Extracts

**Figure 3:**
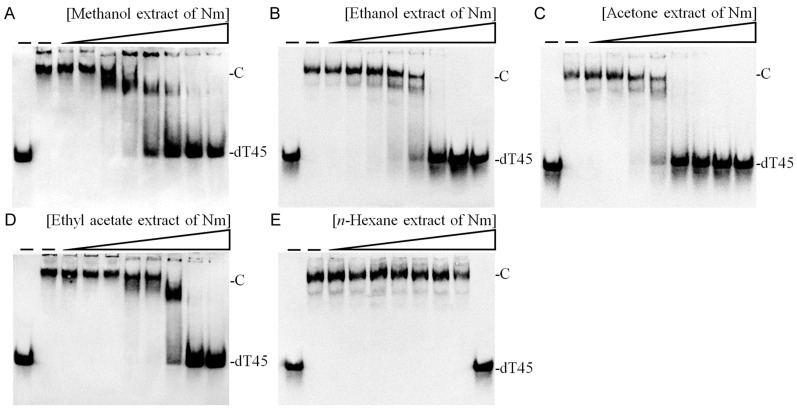
Inhibition assay of Nsp9 ssDNA binding activity using *N. miranda* extracts. Nsp9 (100 μM) was incubated with *N. miranda* leaf extracts prepared with (**A**) methanol, (**B**) ethanol, (**C**) acetone, (**D**) ethyl acetate, and (**E**) *n*-hexane at concentrations ranging from 0 to 1000 μg/mL (0, 7.8, 15.6, 31.3, 62.5, 125, 250, 500, and 1000 μg/mL). Increasing concentrations of extracts led to an observable reduction in ssDNA band shift, indicating disruption of the Nsp9–ssDNA complex formation. Notably, the extracts obtained with methanol, ethanol, and acetone showed significant inhibitory effects on Nsp9 activity.

A primary aim of this research was to investigate the potential of *N. miranda* extracts in inhibiting Nsp9, a protein conserved across SARS-CoV-2 variants, as a step toward novel drug development. After observing no inhibitory effects from known SSB inhibitor myricetin and Nsp9 binder oridonin on Nsp9 activity, our search extended to identifying potential Nsp9 inhibitors from plant extracts. Extracts from *N. miranda* leaves were prepared using six different solvents: distilled water, methanol, ethanol, acetone, ethyl acetate, and *n*-hexane, to assess their inhibitory effects on Nsp9. To date, no plant extracts have been confirmed to inhibit Nsp9 activity. In our inhibitory assays, we tested various concentrations (0–1000 μg/mL) of these *N. miranda* leaf extracts. While the water extract showed no effect on Nsp9 activity (Figure 2D), extracts obtained with other solvents had varying impacts on inhibition, depending on the solvent used. Increasing concentrations of extracts led to an observable reduction in ssDNA band shift, indicating disruption of the Nsp9–ssDNA complex formation. Specifically, extracts derived from methanol (Figure 3A), ethanol (Figure 3B), acetone (Figure 3C), ethyl acetate (Figure 3D), and *n*-hexane (Figure 3E) reduced Nsp9 activity by 77%, 90%, 100%, 38%, and 0%, respectively, at a concentration of 250 μg/mL. The calculated IC_50_ values were 103.4 ± 8.2 μg/mL for methanol, 138.9 ± 12.2 μg/mL for ethanol, 72.5 ± 6.0 μg/mL for acetone, 307.7 ± 20.4 μg/mL for ethyl acetate, and 775.4 ± 30.2 μg/mL for *n*-hexane extracts, respectively (Table 1). An inhibitory assay was also conducted with acetone extract from *Sinningia bullata* leaves, which showed no inhibitory effect on Nsp9. Thus, specific compounds within the *N. miranda* leaf extracts, particularly those extracted with methanol, ethanol, and acetone, may function independently or in synergy to effectively inhibit Nsp9.

### 2.4. Molecular Docking of Nsp9

**Figure 4:**
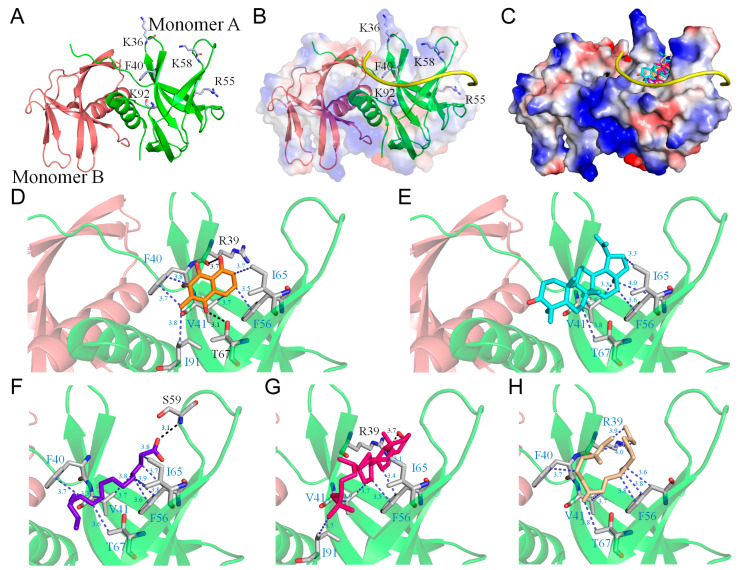
Molecular docking analysis of Nsp9. (**A**) The crystal structure of the Nsp9 dimer (PDB ID 6WXD), with monomers A and B colored differently. Key nucleic acid-binding residues K36, F40, R55, K58, and K92 are labeled for clarity. (**B**) A hypothetical model of the Nsp9–RNA complex, constructed based on the assumption that residues K36, F40, R55, K58, and K92 are crucial for nucleic acid binding. The RNA modeled in the complex is highlighted in yellow. (**C**) Docking analysis illustrating the five most abundant compounds from the *N. miranda* leaf extract individually docked into Nsp9: plumbagin (orange), lupenone (cyan), palmitic acid (purple-blue), stigmast-5-en-3-ol (hot pink), and neophytadiene (wheat). These compounds interact with RNA binding sites, occupying the cavity of the RNA-binding surface. Charge distribution patterns are shown to delineate RNA binding sites for enhanced clarity. (**D**–**H**) depict the binding modes of plumbagin, lupenone, palmitic acid, stigmast-5-en-3-ol, and neophytadiene, respectively, to Nsp9. Each compound binds to Nsp9 with unique poses and at different sites. Residues involved in hydrogen bonding are marked in black, while those contributing to hydrophobic interactions are shown in blue.

Among the various extracts tested, the acetone extract from *N. miranda* leaves showed the most effective inhibition of Nsp9 (Table 1). Previous analyses [60] using gas chromatography–mass spectrometry (GC–MS) have identified five major compounds, each representing more than 4% of the extract composition, specifically plumbagin (28.52%), lupenone (11.45%), palmitic acid (5.49%), stigmast-5-en-3-ol (5.06%), and neophytadiene (4.72%). These compounds are speculated to play a role in Nsp9 inhibition. The crystal structure of the Nsp9 dimer (PDB ID 6WXD) [26] provides a foundation (Figure 4A) for molecular docking studies to identify potential binding sites and assess binding energies for drug development. Although the complex structure of Nsp9 with a ssDNA or RNA polymer has not been characterized, preventing the precise identification of the nucleic acid binding site, nuclear magnetic resonance spectroscopy (NMR) combined with surface biolayer interferometry (BLI) has identified a binding interface for ssDNA and RNA [61]. We constructed a hypothetical Nsp9–RNA complex model, highlighting residues K36, F40, R55, K58, and K92 as crucial for nucleic acid binding (Figure 4B). Using AutoDock Vina (Figure 4C), the top five compounds, namely, plumbagin (Figure 4D), lupenone (Figure 4E), palmitic acid (Figure 4F), stigmast-5-en-3-ol (Figure 4G), and neophytadiene (Figure 4H) from the acetone extract, were individually docked to Nsp9, and it was found that they all targeted areas associated with RNA interaction. These compounds interacted with RNA binding sites, occupying the cavity of the RNA-binding surface and engaging in hydrophobic interactions with several key residues, including R39, F40, V41, F56, I65, T67, and I91 (Table 2). Among these residues, F40 in Nsp9 is known as a critical residue for nucleic acid binding. In addition, some compounds formed hydrogen bonds with critical RNA-interacting residues, suggesting a collective potential to impede RNA’s binding to Nsp9. For example, plumbagin and stigmast-5-en-3-ol formed hydrogen bonds with R39 (3.7 Å), while palmitic acid potentially bonded to S59 (3.1 Å). Crucial RNA-binding residues, K58 and K92, were also found within contact distance (< 4 Å) to the compounds, indicating potential interactions. The binding affinities of these compounds suggest a hierarchy in their effectiveness, with stigmast-5-en-3-ol showing the highest potential. The binding efficiency of these compounds was ranked as follows: stigmast-5-en-3-ol > lupenone > plumbagin > neophytadiene > palmitic acid (Table 2). This diverse interaction pattern at RNA-binding sites implies that the leaf extract’s inhibitory action on Nsp9 could result from a synergistic effect among these compounds. However, this speculation requires further biochemical and structural validation. Our laboratory is currently exploring these interactions through complex crystal structure analysis.

### 2.5. Cytotoxic Effects of N. miranda Extract on H1975 Lung Carcinoma Cells

**Figure 5:**
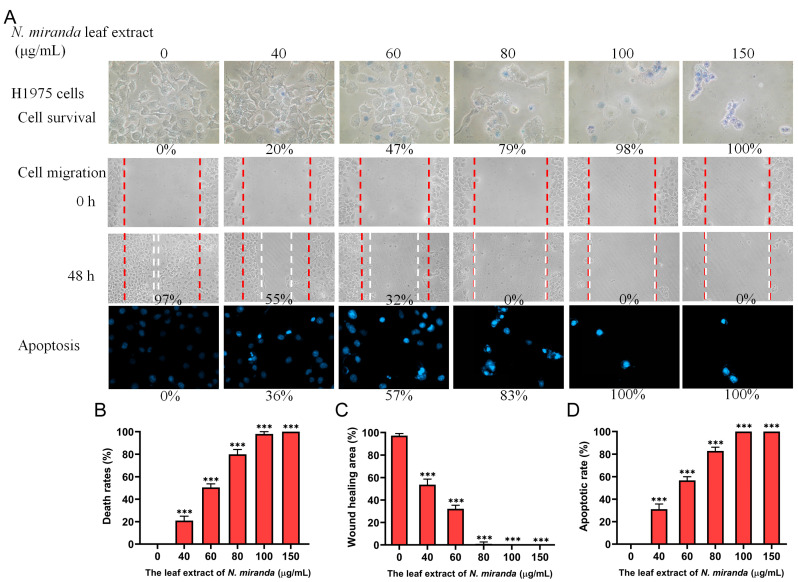
Anticancer potential of *N. miranda* leaf extract on H1975 lung carcinoma cells. (**A**) The effect of *N. miranda* extract on cell survival, migration, and nuclear condensation in H1975 cells. (**B**) Trypan blue exclusion assay results demonstrating cell viability post-exposure to varying concentrations of *N. miranda* extract. (**C**) Wound-healing assay results showing the migration of H1975 cells before and 48 h after treatment with the extract at different concentrations. (**D**) Hoechst staining results depicting the levels of apoptosis and DNA fragmentation across a range of *N. miranda* extract concentrations. Statistical significance relative to the control is indicated by *** for *p* < 0.001.

Exploring the potential of *N. miranda* leaf extract beyond its inhibitory effects on Nsp9 and its potential advantages in curbing SARS-CoV-2 replication, we extended our investigation to assess its effects on various lung carcinoma cells. The methanol extract of *N. miranda* was selected for its anticancer properties, specifically against H1975 cells, to examine its efficacy in inducing cytotoxicity, inhibiting cell migration, and triggering apoptosis (Figure 5A). Originating from a non-smoking female diagnosed with non-small cell lung cancer (NSCLC), the H1975 cell line exhibits epithelial morphology and harbors significant mutations in the epidermal growth factor receptor (EGFR) and TP53 genes. This includes the EGFR exon 21 L858R mutation, which is known to enhance sensitivity to EGFR inhibitors, a common treatment for NSCLC [62]. To evaluate cytotoxicity, we employed the trypan blue staining assay, which is predicated on the principle that non-viable cells absorb the dye due to compromised membranes, thus facilitating the differentiation between viable and non-viable cells. H1975 cell cultures in 96-well plates were treated with varying concentrations of the extract. The extract stock (20 mg/mL) was diluted with a culture medium to achieve the desired concentrations, and cells were incubated with these dilutions or with a culture medium containing 0.2% DMSO as a control. The assay revealed significant cytotoxic effects of the *N. miranda* extract on H1975 cells, with cell mortality increasing in a dose-dependent manner when treated with concentrations of 0, 40, 60, 80, 100, and 150 μg/mL, leading to mortality rates of 0%, 20%, 47%, 79%, 98%, and 100%, respectively (Figure 5B). We also examined the cytotoxic effect using 3-(4,5-dimethylthiazol-2-yl)-2,5-diphenyltetrazolium bromide (MTT). Through MTT assay, *N. miranda* leaf extract was found to significantly inhibit H1975 cell growth in a dose-dependent manner with an IC_50_ value of 69.8 ± 3.6 μg/mL. Consequently, these results from trypan blue staining and MTT assays indicated the anticancer potential of *N. miranda* leaf extract. The marked difference in survival between treated and control groups highlights the potent cytotoxic capability of the *N. miranda* extract, suggesting that its active compounds are highly effective in inducing cancer cell death.

### 2.6. Inhibition of H1975 Cell Migration by N. miranda Extract

The *N. miranda* extract demonstrated significant suppressive impacts on the migration of H1975 lung cancer cells, addressing a key factor in cancer metastasis. The effectiveness of the extract was evaluated through a wound-healing assay, a dependable laboratory technique for assessing cellular migration within a two-dimensional setup. In this procedure, a wound was created within a dense cell layer, and different concentrations of *N. miranda* extract were then applied. This created gap served as a cue for surrounding cells to migrate and close the gap. Observations made immediately and 48 h post-treatment revealed a pronounced decrease in migration rate with increasing concentrations of the extract. Specifically, treatment with 0, 40, 60, 80, 100, and 150 μg/mL of *N. miranda* extract led to wound closure percentages of 97%, 55%, 32%, 0%, 0%, and 0%, respectively (Figure 5C). The observed reduction in cell movement, which became statistically significant with higher doses of the extract, suggests a potent antimetastatic property of the extract. Notably, cell migration was completely inhibited at doses of 80 μg/mL and higher, highlighting the extract’s capacity to prevent the spread of cancer cells. Thus, *N. miranda* extract holds promise for applications aimed at reducing cancer metastasis.

### 2.7. Induction of Apoptosis in H1975 Cells by N. miranda Extract

Our study reveals that the *N. miranda* extract triggers apoptosis in H1975 cells, as detected through Hoechst staining. The dye Hoechst 33342 used in this assay selectively penetrates cells, staining all cell nuclei. This dye is notably more attracted to the condensed chromatin in apoptotic cells, leading to a brighter fluorescence than that observed in non-apoptotic cells. The degree of DNA fragmentation, a definitive indicator of apoptosis, increased significantly with the extract dosages of 0, 40, 60, 80, 100, and 150 μg/mL, showing increments of 0%, 36%, 57%, 83%, 100%, and 100% in DNA fragmentation, respectively (Figure 5D). As the concentration of the *N. miranda* extract increased, so did the appearance of apoptosis indicators such as chromatin condensation and nuclear fragmentation, signifying a concentration-dependent increase in apoptotic death. These signs of apoptosis were identified by the enhanced blue fluorescence from the Hoechst dye upon binding to apoptotic nuclei. Achieving full apoptosis with extract concentrations of 100 μg/mL and up underscores the extract’s potent apoptotic effect. Consequently, the *N. miranda* extract contains active components capable of activating specific apoptosis pathways, shedding light on how the extract works and its potential application in cancer treatment strategies.

### 2.8. G2 Cell-Cycle Arrest in H1975 Cells by N. miranda Leaf Extract

**Figure 6:**
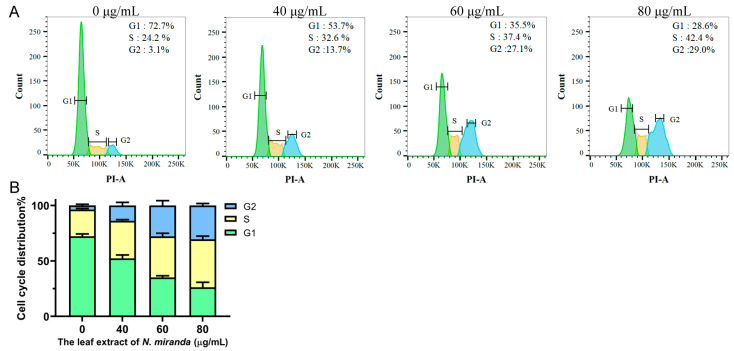
(**A**) Alteration of cell cycle progression by *N. miranda* leaf extract in H1975 cells. H1975 cells underwent treatment with a control solution (0.1% DMSO) or with *N. miranda* leaf extract at specified concentrations for 24 h and were subsequently fixed in 70% ethanol overnight. The cells were then stained with propidium iodide (PI) for 30 min before analysis via flow cytometry. (**B**) The cell cycle distribution.

Flow cytometry was employed to evaluate the cell cycle progression in H1975 cells after exposure to *N. miranda* leaf extract (Figure 6A). When cells were treated with the extract at concentrations of 40, 60, and 80 µg/mL, there was a notable increase in the proportion of cells in the G2 phase—from 3.1% in control groups to 13.7%, 27.1%, and 29.0% in treated groups, respectively—highlighting a dose-dependent G2 phase accumulation (Figure 6B). Such accumulation suggests a significant alteration in the typical cell cycle progression, critical for cellular division. The observed increase in cells at the G2 phase points toward the *N. miranda* leaf extract’s potential to mediate its anticancer effects by triggering a halt in the G2 phase, effectively reducing the rate of cell multiplication in these carcinoma cells.

### 2.9. Anticancer Potential of N. miranda Extract on A549 Lung Carcinoma Cells

**Figure 7:**
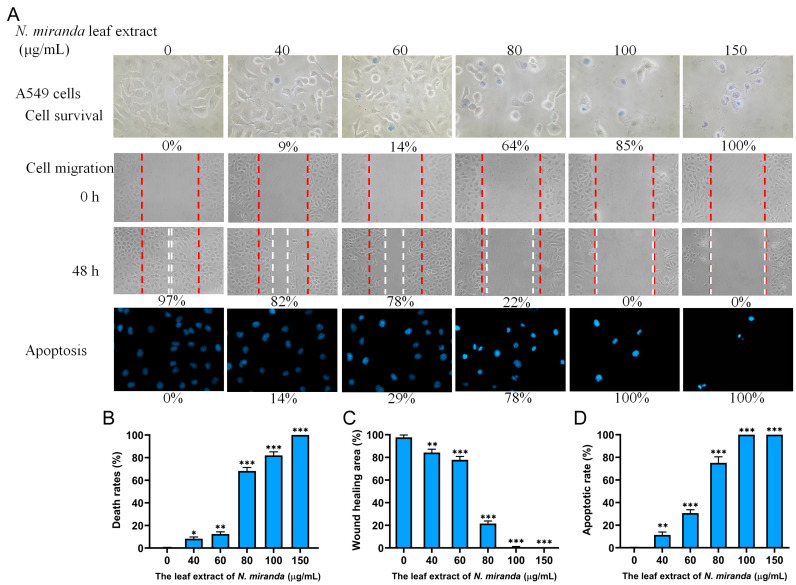
Anticancer potential of *N. miranda* leaf extract on A549 lung carcinoma cells. (**A**) The effect of *N. miranda* extract on cell survival, migration, and nuclear condensation in A549 cells. (**B**) Trypan blue exclusion assay results demonstrating cell viability post-exposure to varying concentrations of *N. miranda* extract. (**C**) Wound-healing assay results showing the migration of A549 cells before and 48 h after treatment with the extract at different concentrations. (**D**) Hoechst staining results depicting the levels of apoptosis and DNA fragmentation across a range of *N. miranda* extract concentrations. Statistical significance relative to the control is indicated by * for *p* < 0.05, ** for *p* < 0.01, and *** for *p* < 0.001.

Building on the positive outcomes with H1975 cells, we further explored the efficacy of *N. miranda* leaf extract against other types of human lung cancer cells, focusing on A549 cells to evaluate the extract’s cytotoxicity, anti-migration, and apoptosis-inducing effects (Figure 7A). A549 cells, characterized as adenocarcinomic human alveolar basal epithelial cells, were derived from the lung tissue of a 58-year-old Caucasian male diagnosed with lung cancer [63]. The administration of the extract at varying concentrations—0, 40, 60, 80, 100, and 150 µg/mL—resulted in corresponding cell mortality rates of 0%, 9%, 14%, 64%, 85%, and 100%, respectively (Figure 7B). The wound-healing assays revealed wound closures of 97%, 82%, 78%, 22%, 0%, and 0% at these same concentrations, indicating a significant inhibitory effect on cell migration (Figure 7C). DNA fragmentation analysis further confirmed the pro-apoptotic influence of the extract, with increases of 0%, 14%, 29%, 78%, 100%, and 100% in DNA fragmentation at the respective concentrations (Figure 7D). Consequently, A549 cells also exhibited susceptibility to the anticancer properties of *N. miranda* extract.

### 2.10. Anticancer Potential of N. miranda Extract on H838 Lung Carcinoma Cells

**Figure 8:**
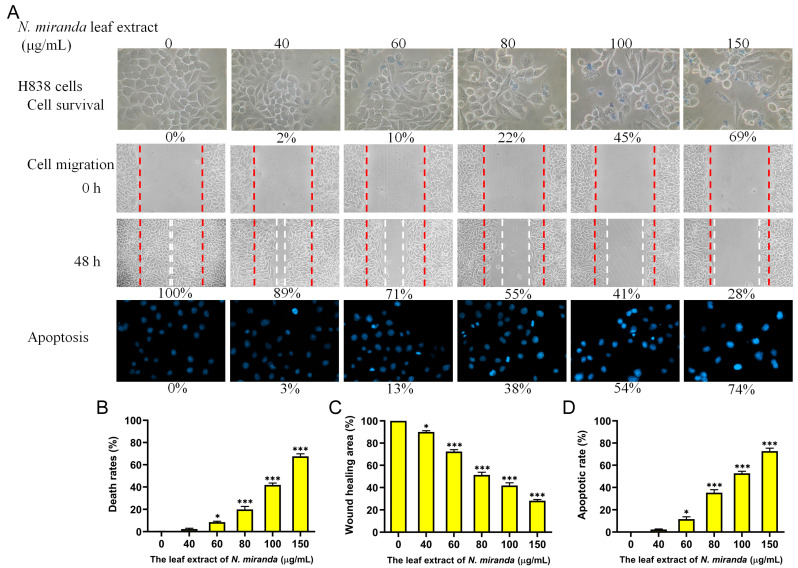
Anticancer potential of *N. miranda* leaf extract on H838 lung carcinoma cells. (**A**) The effect of *N. miranda* extract on cell survival, migration, and nuclear condensation in H838 cells. (**B**) Trypan blue exclusion assay results demonstrating cell viability post-exposure to varying concentrations of *N. miranda* extract. (**C**) Wound-healing assay results showing the migration of H838 cells before and 48 h after treatment with the extract at different concentrations. (**D**) Hoechst staining results depicting the levels of apoptosis and DNA fragmentation across a range of *N. miranda* extract concentrations. Statistical significance relative to the control is indicated by * for *p* < 0.05 and *** for *p* < 0.001.

The anticancer efficacy of *N. miranda* extract was further assessed on H838 lung carcinoma cells (Figure 8A). The H838 cell line, originating from the lung tissue of a 59-year-old Caucasian male diagnosed with stage 3B adenocarcinoma, exhibits epithelial characteristics [64]. Treatments with the extract at concentrations of 0, 40, 60, 80, 100, and 150 μg/mL resulted in cell mortality rates of 0%, 2%, 10%, 22%, 45%, and 69%, respectively (Figure 8B). Wound closure measurements at these concentrations showed reductions of 100%, 89%, 71%, 55%, 41%, and 28%, respectively, indicating a significant effect on cell migration (Figure 8C). DNA fragmentation analysis further showed increases of 0%, 3%, 13%, 38%, 54%, and 74%, respectively, indicating the extract’s ability to induce apoptosis in H838 cells (Figure 8D). Thus, the extract of *N. miranda* demonstrated a notable anticancer effect on H838 cells as well.

### 2.11. Comparative Anticancer Effects on Lung Carcinoma Cells

In this study, we observed notable inhibitory effects of the methanol extract of *N. miranda* leaves on lung cancer cell survival, migration, and the induction of apoptosis. The degree of effectiveness varied among the cell lines tested. From the combined data of the trypan blue staining assays, the IC_50_ values were determined to be 61.7 ± 2.1 μg/mL for H1975 cells, 74.7 ± 3.0 μg/mL for A549 cells, and 110.4 ± 4.8 μg/mL for H838 cells. Thus, the anticancer efficacy of the *N. miranda* extract was ranked as follows: H1975 > A549 > H838. This suggests that although all the tested cell lines are forms of lung carcinoma, the extract of *N. miranda* may exhibit specificity toward them, with varying degrees of effectiveness.

### 2.12. Comparative Analysis of Anticancer Potentials of N. miranda Extracts Obtained Using Various Solvents

**Figure 9:**
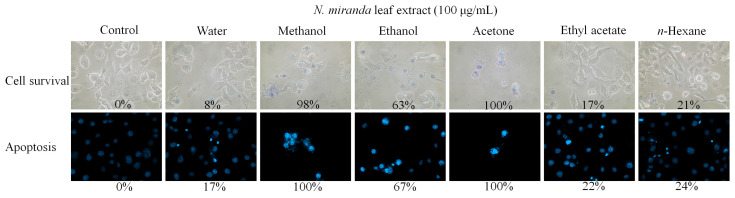
Anticancer potential of *N. miranda* leaf extract against H1975 lung carcinoma cells, based on solvent effects. The effect of extracts prepared using different solvents—distilled water, methanol, ethanol, acetone, ethyl acetate, and *n*-hexane—on cell survival and nuclear condensation, as assessed through trypan blue exclusion assay and Hoechst staining was examined. Extracts obtained with acetone and methanol demonstrated the highest anticancer potential among the tested solvents.

The methanol extract’s anticancer activity might be attributed to specific compounds it contains. To delve deeper into the effectiveness of solvent extraction, we compared extracts obtained using various solvents in their ability to inhibit cancer cell growth and induce apoptosis in H1975 cells (Figure 9). At a concentration of 100 μg/mL, extracts derived from distilled water, methanol, ethanol, acetone, ethyl acetate, and *n*-hexane led to 8%, 98%, 63%, 100%, 17%, and 21% cell mortality in H1975 cells, respectively, and triggered apoptosis in 17%, 100%, 67%, 100%, 22%, and 24% of cells, correspondingly. Consequently, the effectiveness of the *N. miranda* extracts, based on the solvent used for extraction, was determined as follows: acetone > methanol > ethanol > *n*-hexane > ethyl acetate > distilled water. This sequence highlights the significant role of the solvent choice in the extraction process on the anticancer properties of the extracts.

### 2.13. Combined Effect of N. miranda Leaf Extract and the Clinical Anticancer Drug Afatinib on H1975 Cells

**Figure 10:**
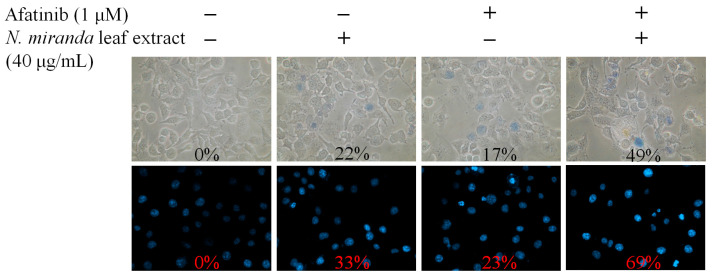
Synergistic effects of *N. miranda* leaf extract and afatinib on H1975 cells. The combined effect of *N. miranda* leaf extract (40 μg/mL) and afatinib (1 μM) on cell survival and apoptosis in H1975 cells was illustrated. Cell viability was assessed using trypan blue exclusion staining, and apoptosis was detected through Hoechst staining. The results suggest that combining afatinib with *N. miranda* leaf extract may enhance therapeutic efficacy against lung cancer.

We further investigated the synergistic effect of combining *N. miranda* leaf extract, prepared using methanol, with the clinical anticancer drug afatinib on H1975 carcinoma cells (Figure 10). Afatinib [65], marketed as Gilotrif, among other names, is an aniline-quinazoline derivative and part of the tyrosine kinase inhibitor family, typically administered orally for treating specific NSCLC cases with EGFR mutations. Previous studies have shown afatinib’s synergistic potential with various agents like aspirin [66], vinorelbine [67], and su11274 [68] in lung cancer therapy. Consequently, our experiment explored the enhanced therapeutic impact of afatinib in conjunction with *N. miranda* leaf extract against H1975 cells. Utilizing a methanol-extracted *N. miranda* leaf extract concentration of 40 μg/mL, which alone has minimal cytotoxic effects, 1 μM afatinib, and the co-treatment with the extract and afatinib resulted in H1975 cell death rates of 22%, 17%, and 49%, and apoptosis rates of 33%, 23%, and 69%, respectively. These outcomes indicate a synergistic cytotoxic effect, with the combined treatment causing higher rates of cell death and DNA fragmentation in H1975 cells than either treatment alone. This suggests that afatinib, when used in combination with *N. miranda* leaf extract, could enhance cancer treatment efficacy. However, further experimental and clinical investigations are necessary to confirm these synergistic effects.

### 2.14. Gas Chromatography–Mass Spectrometry (GC–MS) Analysis

In light of the anti-Nsp9 and anticancer activities of *N. miranda* leaf extract prepared using methanol, we conducted gas chromatography–mass spectrometry (GC–MS) analysis to identify the predominant compounds within the extract. The spectral data generated were compared with the NIST 2011 and Wiley 10th edition mass spectral libraries, allowing for the tentative identification of the compounds. Compounds with a similarity index (SI) greater than 800 were considered for identification. Accordingly, the top 19 compounds, each constituting more than 0.5% of the extract, were identified as follows: 13-docosenamide (17.4%), hexadecanoic acid (15.9%), octadecanoic acid (8.5%), neophytadiene (6.9%), vitamin E (5.7%), hexadecanoic acid, methyl ester (5.1%), 9,12,15-octadecatrienoic acid, methyl ester (3.8%), stigmast-5-en-3-ol (3.6%), lupeol (3.1%), lup-20(29)-en-3-one (3.0%), 9,12-octadecadienoic acid (2.3%), 3,7,11,15-tetramethyl-2-hexadecen-1-ol (2.1%), methyl stearate (1.8%), 2,4-di-tert-butylphenol (1.6%), hexadecanoic acid, 2-hydroxy-1-(hydroxymethyl)ethyl ester (1.1%), 2-cyclohexen-1-one, 4-(3-hydroxybutyl)-3,5,5-trimethyl- (0.9%), methyl 11-docosenoate (0.8%), stigmasta-3,5-diene (0.6%), and 9-octadecenoic acid (0.5%). These results provide a molecular basis for further drug development, suggesting that one or more of these compounds in the 100% methanol extract of *N. miranda* leaf may act as potential anti-lung cancer and anti-Nsp9 agents, either individually or synergistically.

## 3. Discussion

Plant carnivory is an evolutionary adaptation that enables plants to thrive in nutrient-deficient environments by providing an alternative means to acquire essential nutrients such as nitrogen and phosphorus [69]. The genus *Nepenthes*, which includes nearly 120 species, represents some of the most extensively studied carnivorous pitcher plants [4,70]. These plants are not only noteworthy for their unique ecological adaptations but also for their various ethnobotanical applications worldwide [1,4,71]. Due to their long history in traditional medicine, *Nepenthes* extracts are considered relatively safe for pharmaceutical use, typically exhibiting minimal side effects in humans. For instance, Malaysian tribes traditionally use boiled roots of *N. ampullaria* and *N. gracilis* to treat stomachaches, while infused parts of their stems are used to reduce fever [4]. Exploring the drug-like properties of *Nepenthes* leaf extracts is particularly significant. Leaf extracts may provide a viable alternative to root or stem extracts. Leaves are more abundantly available than roots or stems, enhancing the sustainability of extract production. Moreover, harvesting leaves does not compromise the survival of the plant as drastically as removing the roots or stems, which can be lethal. Thus, utilizing leaf extracts not only preserves plant life but also ensures more sustainable extraction practices. Given these advantages, the leaf extract of *Nepenthes*, particularly that of *N. miranda* as investigated in this study, is recommended for further research into its pharmaceutical efficacy and potential for broad medical applications.

Cancer remains a major cause of mortality worldwide [72,73], with cancer cells adept at co-opting and modifying metabolic pathways to support their survival and proliferation, complicating treatment efforts [74,75]. Traditional cancer therapies typically involve radiotherapy and chemotherapy, which are often associated with severe side effects and can lead to additional health complications [39,40]. Consequently, there is increasing interest in exploring natural compounds as potential anticancer agents, both in vitro and in vivo [76,77,78]. Prominent examples of plant-derived anticancer medications include vincristine, vinblastine, and paclitaxel, which have been successfully integrated into clinical practice [79,80]. The discovery of additional plant-based compounds for pharmaceutical use remains a priority. In this study, the leaf extract of *N. miranda* has demonstrated potential anticancer properties by inhibiting cell survival and migration and inducing apoptosis in various pulmonary carcinoma cell lines such as H1975 (Figure 5), A549 (Figure 7), and H838 (Figure 8). Notably, *N. miranda* leaf extract exhibited potent effects in reducing viability and migration and triggering apoptosis across these cell lines. Specific cytotoxic activities were quantified, yielding IC_50_ values of 61.7 ± 2.1 μg/mL for H1975 cells, 74.7 ± 3.0 μg/mL for A549 cells, and 110.4 ± 4.8 μg/mL for H838 cells, with a pronounced sensitivity in H1975 cells (Figure 5). This order of efficacy (H1975 > A549 > H838) highlights a potential specificity of the extract toward the H1975 cell line, which harbors critical mutations in the EGFR and TP53 genes [62], including an EGFR exon 21 L858R mutation known to confer increased sensitivity to EGFR inhibitors—a staple in NSCLC treatment. These findings suggest that *N. miranda* leaf extract could serve as a complementary or alternative therapeutic option in managing NSCLC. Moreover, combining this extract with the clinical anticancer drug afatinib could enhance therapeutic outcomes due to synergistic effects (Figure 10). Our results advocate for the integration of *N. miranda* leaf extract into lung cancer treatment regimens and underscore the need to isolate and identify the active components within the extract to optimize therapeutic formulations and explore their pharmacological potentials further.

NSCLC is the predominant cause of cancer-related mortality globally, with approximately 17% of patients presenting with activating mutations in the EGFR gene [81]. The most common mutations include Del19 and L858R, which serve as positive predictive markers for the efficacy of EGFR tyrosine kinase inhibitors (TKIs). Currently, various EGFR-TKIs, including afatinib, are standard treatments for patients with these activating mutations. Afatinib, a second-generation TKI, is effective not only against mutations targeted by first-generation TKIs such as erlotinib and gefitinib but also against rarer mutations resistant to these earlier drugs [82]. There is also growing evidence supporting afatinib’s utility in treating other EGFR and Her2-driven cancers, including breast cancer [83]. However, afatinib does not target the T790M mutation, which commonly confers resistance [81]. Resistance to EGFR-TKIs remains a significant challenge in managing EGFR-mutated NSCLC effectively; hence, developing novel therapeutics to overcome resistance is still crucial and could tailor treatment strategies to improve patient quality of life and survival [81]. In this study, *N. miranda* leaf extract demonstrated significant anticancer effects, reducing cell viability, migration, and proliferation and inducing apoptosis in NSCLC cell lines (Figure 5, Figure 6, Figure 7 and Figure 8). Additionally, its anticancer efficacy was found to be synergistically enhanced when used in conjunction with afatinib (Figure 10). Various valuable metabolites identified in different *Nepenthes* species, notably naphthoquinones such as plumbagin from *N. alata*, have shown significant anticancer activities [3,84]. Plumbagin, in particular, has reduced tumor growth in animal models without adverse effects [85] and has been shown to be effective against breast and pancreatic cancers in both in vivo and in vitro settings [86,87]. Our previous studies on the complex crystal structure of plumbagin have elucidated its mechanism of inhibiting dihydroorotase [5], a crucial enzyme in pyrimidine biosynthesis [88,89,90,91], underscoring its potential in chemotherapy. The leaf extract of *N. miranda* (Table 2), rich in compounds like lupenone [92,93], palmitic acid [94], stigmast-5-en-3-ol [95], and neophytadiene [96,97], may collectively exhibit potent cytotoxic effects due to their known anticancer properties. Further investigation is needed to explore the polypharmacological and synergistic potentials of these compounds and to determine optimal ratios for effective cancer treatment. In addition, our flow cytometry analysis suggests that *N. miranda* leaf extract might inhibit the proliferation of H1975 cells by inducing G2 phase cell-cycle arrest, underscoring the critical role of the G2 checkpoint as a genomic guardian in tumor cells [98]. Targeting cell cycle proteins and disrupting this checkpoint is emerging as a promising anticancer strategy [98]. Our ongoing research focuses on delineating the specific cellular pathways leading to G2 arrest in these cells, with the aim of advancing the development of novel NSCLC therapies.

SARS-CoV-2, a member of the beta-coronaviruses, encodes four structural proteins—spike, envelope, membrane, and nucleoprotein—and 16 nonstructural proteins [99]. The structural proteins are crucial for virion assembly and modulating the host immune response, whereas the nonstructural proteins primarily form the replication–transcription complex, facilitating viral RNA replication and translation [9]. While significant advancements have been made in detecting SARS-CoV-2 through enzyme-linked aptamer-antibody sandwich assays and hybrid lateral flow strips [100], there remains a critical need to target viral nonstructural proteins [101] to develop new therapeutic interventions [102]. Current strategies against COVID-19 include vaccines, neutralizing antibodies, and antiviral drugs [103]. Despite progress in vaccine and antibody development, the numerous N-linked glycosylation sites on the SARS-CoV-2 spike protein pose challenges in maintaining the effectiveness of these approaches [11,99,104,105]. Consequently, pharmacotherapy could serve as a complementary strategy to vaccines [20]. For instance, Nirmatrelvir, a main protease inhibitor (Nsp5) found in Paxlovid, has been approved for treating high-risk COVID-19 patients [16], though resistance to this drug has been reported shortly after its introduction [17,18]. There is an ongoing need to develop new antiviral agents and explore complementary and alternative medicines to address the evolving threat of SARS-CoV-2 [13,14,15,19,20]. Given its essential and conserved nature in various SARS-CoV-2 strains (Figure 1), Nsp9 presents a promising new target for drug discovery [24,106], warranting further investigation beyond existing protease inhibitors.

Nsp9 is a conserved coronaviral replicase essential for priming viral RNA synthesis and acts as a critical component of the multi-subunit viral replication/transcription complex [21,22,23,24,25]. The enzymatic activity of the SARS-CoV-2 NiRAN domain is crucial for viral propagation, with three distinct activities associated with modification of the Nsp9 N terminus, NMPylation, RNAylation, and deRNAylation/capping [21,24]. During viral RNA synthesis, Nsp9 serves as an essential substrate for forming a covalent RNA–protein intermediate, thus positioning it as a promising target for developing interventions that could potentially suppress SARS-CoV-2 infection [107,108,109]. Nsp9’s capacity to bind RNA suggests it may be a viable druggable target. Notably, Nsp9 is also proficient in binding ssDNA (Figure 2), similar to its affinity for RNA [26]. The crystal structure of Nsp9 exhibits an OB fold-like structure [26], which is characteristic of SSBs that bind to ssDNA [110]. Leveraging this structural similarity between SSB and Nsp9, we explored the potential of SSB inhibitors to suppress Nsp9’s nucleic acid binding activity (Figure 3). In this study, *N. miranda* leaf extract demonstrated an ability to inhibit this activity, whereas another SSB inhibitor, myricetin, did not affect Nsp9 (Table 1). Future research should focus on identifying specific components of the *N. miranda* extract that inhibit Nsp9’s nucleic acid binding activity, providing a basis for targeted drug development against SARS-CoV-2.

Screening of a natural product library to identify Nsp9 binders via native mass spectrometry led to the discovery of oridonin, which binds to Nsp9 with micromolar affinities [27,111,112,113]. Oridonin’s interaction with Nsp9 inhibits its ability to act as a substrate for the NiRAN domain, demonstrating its potential as a druggable target to suppress SARS-CoV-2 infection [27]. The crystallographic analysis of the Nsp9-oridonin complex reveals that oridonin binds at a conserved site near the C-terminal GxxxG-helix of Nsp9 [27]. According to our docking studies (Figure 4), this site does not overlap with the binding sites of major components from *N. miranda* leaf extract, such as plumbagin, lupenone, palmitic acid, stigmast-5-en-3-ol, and neophytadiene. Although oridonin does not inhibit the nucleic acid binding activity of Nsp9 (Figure 2), it is worth investigating whether active components in *N. miranda* leaf extract could synergize with oridonin to target the multifunctional roles of Nsp9. Given the critical need for new inhibitors and alternative medicines that target this conserved viral protein, our laboratory is currently advancing this research by producing co-crystals of these compounds with purified Nsp9, aiming to obtain complexed crystal structures that could facilitate optimized drug design and development.

Despite the lack of a complexed structure of Nsp9 with ssDNA/RNA, a binding interface for these nucleic acids has been proposed [61]. Our docking studies (Figure 4) indicate that five compounds found in the *N. miranda* extract—plumbagin, lupenone, palmitic acid, stigmast-5-en-3-ol, and neophytadiene—target various ssDNA binding sites on Nsp9, suggesting a potential collective mechanism that could impede the binding of ssDNA to Nsp9. The binding efficiency of these compounds was ranked as follows: stigmast-5-en-3-ol > lupenone > plumbagin > neophytadiene > palmitic acid (Table 2). Thus, the effectiveness of N. miranda’s leaf extract in inhibiting Nsp9 could be attributed to the synergistic actions of these compounds, particularly the potential inhibitory effects of stigmast-5-en-3-ol and lupenone. However, these assumptions about the collective inhibitory activity of the compounds require further experimental validation to confirm their precise interactions and synergistic effects on Nsp9.

While this study has elucidated the potential applications of *N. miranda*’s leaf extract, it is important to note that these effects are dependent on the extraction solvent used. For Nsp9 inhibition, the IC_50_ values are as follows: methanol extract at 103.4 ± 8.2 μg/mL, ethanol extract at 138.9 ± 12.2 μg/mL, acetone extract at 72.5 ± 6.0 μg/mL, ethyl acetate extract at 307.7 ± 20.4 μg/mL, and *n*-hexane extract at 775.4 ± 30.2 μg/mL (Table 1). These results align with the extract’s anticancer potential against H1975 cells. At a concentration of 100 μg/mL, the extracts from distilled water, methanol, ethanol, acetone, ethyl acetate, and *n*-hexane induced apoptosis in 17%, 100%, 67%, 100%, 22%, and 24% of the cells, respectively (Figure 9). Therefore, the efficacy of the *N. miranda* extracts in inhibiting Nsp9 and inducing apoptosis in H1975 cells was determined to be in the following order: acetone, methanol > ethanol > *n*-hexane, ethyl acetate > distilled water. This ranking underscores the critical influence of solvent selection on the biological activity of the extracts.

In conclusion, we evaluated the anti-Nsp9 activity of *N. miranda* leaf extracts obtained using various solvents including distilled water, methanol, ethanol, acetone, ethyl acetate, and *n*-hexane. We assessed the cytotoxic effects of the leaf extract on human NSCLC cell lines H1975, A549, and H838, examining parameters such as cell survival, apoptosis, and migration. Notably, the *N. miranda* leaf extract appears to inhibit the proliferation of H1975 cells by inducing G2 phase cell-cycle arrest. Furthermore, when combined with the clinical anticancer drug afatinib, the extract demonstrated enhanced therapeutic efficacy, likely due to synergistic effects. These results significantly broaden the potential medical applications of *N. miranda* leaf extract, suggesting its use not only in traditional remedies but also as a prospective treatment for pulmonary diseases. This study underscores the pharmacological potential of *N. miranda*, paving the way for further research into its utility in developing innovative therapies for NSCLC.

## 4. Materials and Methods

### 4.1. Materials

All solvents and chemicals were of the highest grade and commercially obtained from Sigma-Aldrich (St. Louis, MO, USA). The *Escherichia coli* strain BL21(DE3) pLysS (Novagen, Worcestershire, UK) was used for recombinant protein expression [114]. The H1975, A549, and H838 cell lines were kindly provided by Dr. Kuo-Ting Chang (Tao Yuan General Hospital). These cells were maintained in RPMI 1640 supplemented with 10% FBS, 100 units/mL penicillin, and 100 μg/mL streptomycin. Cells were incubated at 37 °C in a 95% air and 5% CO_2_ atmosphere.

### 4.2. Expression and Purification of the Recombinant Protein

The synthesized gene encoding Nsp9 was cloned using forward and reverse primers (with underlining indicating the restriction site) Nsp9-NdeI-N (5′-GGGCATATGAATAATGAGCTTAGTCCT-3′) and Nsp9-XhoI-C (5′-GGGCTCGAGTTGTAGACGTACTGTGGC-3′). These primers facilitated the insertion of the gene into the pET21b vector, resulting in the construction of the expression plasmid pET21b-Nsp9. This resulting plasmid was introduced into *E. coli* BL21 (DE3) cells, which were cultured in LB medium with 250 μg/mL ampicillin until reaching an OD_600_ of 0.9, under vigorous shaking at 37 °C. Recombinant Nsp9 expression was induced with 1 mM isopropyl thiogalactopyranoside (IPTG) for 12 h at 25 °C. Nsp9 was purified from the soluble supernatant by using Ni^2+^-affinity chromatography (HisTrap HP; GE Healthcare Bio-Sciences, Piscataway, NJ, USA), eluted with buffer A (20 mM Tris–HCl, 200 mM imidazole, and 0.5 M NaCl, pH 7.9), and dialyzed against a dialysis buffer (20 mM Tris–HCl and 0.1 M NaCl, pH 7.9). The protein purity remained at >97%, as determined using SDS–PAGE (Mini-PROTEAN Tetra System; Bio-Rad, CA, USA).

### 4.3. Electrophoretic Mobility Shift Analysis (EMSA)

For the analysis of Nsp9 binding, a biotinylated deoxythymidine (dT) homopolymer dT45 was employed as a standard assay substrate. The ssDNA dT45 was biotinylated at its 5′ end. Purified Nsp9 was incubated with this labeled ssDNA (30 fmol/μL) at various concentrations (0, 3, 6, 13, 25, 50, 75, 100, 150, and 200 μM). The electrophoretic mobility shift analysis (EMSA) was performed using the LightShift Chemiluminescent EMSA Kit. Briefly, Nsp9 and the DNA substrate dT45 were mixed and incubated for 60 min at 37 °C in a buffer containing 40 mM Tris–HCl (pH 7.5) and 50 mM NaCl. After adding the dye mixture, the reaction samples were resolved on an 8% native polyacrylamide gel using EMSA. Electrophoresis was conducted at 100 V for 1 h in TBE running buffer. The protein–DNA complexes were then electroblotted onto a positively charged nylon membrane (GE, Boston, MA, USA). Cross-linking of the transferred DNA to the membrane was achieved using a UV-light instrument equipped with 312 nm bulbs, with an exposure time of 10 min. Detection of the DNA was carried out using the streptavidin–horseradish peroxidase conjugate and chemiluminescent substrate (Pierce Biotechnology, Waltham, MA, USA). The [Protein]_50_, representing the concentration of protein that bound 50% of the input DNA, was estimated from the EMSA results.

### 4.4. Nsp9 Inhibition

An inhibition assay was conducted with Nsp9 at a concentration of 100 μM, incubated with both dT45 and various concentrations of *N. miranda* extract (0, 7.8, 15.6, 31.3, 62.5, 125, 250, 500, and 1000 μg/mL). Following EMSA, a titration curve was generated based on the experimental data. The concentration of *N. miranda* extract required to achieve 50% inhibition (IC_50_) of Nsp9 activity was directly determined from this curve using graphical analysis.

### 4.5. Plant Materials and Extract Preparations

The leaf extracts of *N. miranda* were obtained using 100% distilled water, methanol, ethanol, acetone, ethyl acetate, and *n*-hexane. The plant material, procured from Guoguang Flower Market and Taiwan Provincial Flower Marketing Cooperative, was identified by Dr. Zhong-Bao Zhang in December 2020 [6]. The collected samples were dried, cut into small pieces, and then pulverized into a powder. For the extraction process, 1 g of the plant powder was placed into a 250 mL conical flask, to which 100 mL of the solvent was added. The mixture was then shaken on an orbital shaker for 5 h. The resulting extract was filtered through a 0.45 μm filter, and the solvent was subsequently removed using a hot air circulation oven set at 40 °C. The extracts were stored at −80 °C until needed. The extract powder was dissolved in 20% DMSO to prepare a stock solution at a concentration of 20 mg/mL. For anticancer cell assays, the stock solution was diluted with a supplemented culture medium to reach the desired assay concentrations. Cancer cells were then incubated with these extract solutions or with the culture medium containing 0.2% DMSO, serving as the treatment and control groups, respectively. For EMSA, the stock solution was diluted with protein storage buffer (40 mM Tris-HCl, 50 mM NaCl, pH 7.5) to the indicated assay concentrations. The protein was then incubated with these extract solutions or with the storage buffer containing 1% DMSO, also serving as treatment and control groups, respectively.

### 4.6. Trypan Blue Cytotoxicity Assay

Cell death was evaluated using the trypan blue cytotoxicity assay [115]. H1975, A549, and H838 cells (5 × 10^3^) were incubated with the *N. miranda* extract in a 100 μL volume for 24 h to assess the cytotoxic activity of the extract via trypan blue dye exclusion.

### 4.7. Chromatin Condensation Assay

Apoptosis in cancer cells was examined using the Hoechst 33342 staining method [116]. H1975, A549, and H838 cells were seeded in 96-well plates at a density of 5 × 10^3^ cells per well and allowed to adhere for 16 h before incubation with *N. miranda* extract for 24 h. Afterward, cells were washed with PBS and stained with Hoechst dye (1 μg/mL) in the dark for 10 min. Stained cells were imaged using ImageXpress Pico (Molecular Devices, CA, USA) with DAPI filter cubes. Image acquisition and analysis were performed using CellReporterXpress Version 2 software.

### 4.8. Wound-Healing Assay

H1975, A549, and H838 cell migration inhibition was investigated using a wound-healing assay [117], a technique to assess collective cell migration in two dimensions. A cell-free area was created in a confluent cell monolayer, and the presence of this gap induced cell migration to close the wound. These cells were grown in serum-reduced medium for 6 h before a linear wound was created using a pipette tip. After washing with serum-reduced medium, cells treated with *N. miranda* extract for 48 h were assessed for migration ability.

### 4.9. Flow Analysis

Flow cytometry was utilized for cell cycle analysis. H1975 cells, treated with DMSO or *N. miranda* extract for 24 h, were harvested with trypsin. These cells were then washed, resuspended in PBS containing 1% FBS, and fixed with cold ethanol (70%). After another wash, cells were incubated in PBS for 5 min, then stained with a PI/RNase solution (PBS, RNase, and 50 μg/mL PI) for 30 min at 37 °C in the dark. Cell cycle distribution was analyzed using a BD FACSCanto II (BD Biosciences, San Jose, CA, USA) and visualized with FlowJo v10 software (Tree Star, Inc., Ashland, OR, USA).

### 4.10. Binding Analysis Using AutoDock Vina

The interaction between compounds and Nsp9 was analyzed using AutoDock Vina [118,119,120]. Nsp9’s atomic coordinates (PDB ID 6WXD) were obtained from the RCSB PDB database. Pre-docking preparations, including charge assignments and volume measurements, were performed using AutoDockTools. The compounds’ 2D structures were sourced from PubChem and translated to .sdf format. Both ligands and the protein target were then prepared as PDBQT files for docking with AutoDock Vina through the PyRx Virtual Screening Tool. Docking results were visualized using PyMOL v2.2.0 software.

### 4.11. GC-MS Analysis

The composition of the leaf extract of *N. miranda*, obtained through methanol extraction, was tentatively identified using a Thermo Scientific TRACE 1300 Gas Chromatograph coupled with a Thermo Scientific ISQ Single Quadrupole Mass Spectrometer System. The chromatographic separation was achieved on an Rxi-5ms column (30 m × 0.25 mm i.d. × 0.25 μm film). Helium was used as the carrier gas at a flow rate of 1 mL/min, with the oven temperature initially set at 40 °C and subsequently increased to 300 °C. The injection port temperature was maintained at 300 °C. Compounds were detected by a quadrupole mass detector, with ionization achieved via electron ionization. Operational settings included a quadrupole temperature of 150 °C, a source temperature of 300 °C, electron energy of 70 eV, a detector temperature of 300 °C, an emission current multiplier voltage of 1624 V, and an interface temperature of 300 °C. The mass range scanned was from 29 to 650 amu. Relative mass fractions of individual chemical components were calculated using the peak area normalization method. Compounds were tentatively identified by comparing the generated spectra with those in the NIST 2011 and Wiley 10th edition mass spectral libraries. Those with a similarity index (SI) exceeding 800 were considered positively identified and are reported in this study.

### 4.12. MTT Cell Viability Assay

The effect of *N. miranda* leaf extract on the viability of H1975 cells was evaluated by MTT assay [121]. Initially, serial dilutions of *N. miranda* leaf extract (40, 60, 80, 100, and 150 μg/mL) were added to cell culture medium in a 96-well plate with H1975 cells per well to a final volume of 100 μL. After 48 h of incubation, 30 μL of an MTT solution (5 mg/mL MTT in PBS) was added to each well. The treated cells were further incubated with MTT solution for 4 h at 37 °C in the dark. The formed formazan crystals were dissolved in 100 μL of DMSO at 37 °C for 10 min. The data were measured on a plate spectrophotometer at 540 nm. Assays were performed in triplicate.

### 4.13. Statistical Analysis

Experiments were conducted in triplicate, with results presented as mean ± standard deviation (SD). IC_50_ values and statistical significance (*p* < 0.05) were determined using SigmaPlot version 12.0 and GraphPad Prism5 (GraphPad Software Inc., San Diego, CA, USA), respectively, with one-way ANOVA employed to assess differences between means.

## Figures and Tables

**Table 1:** Inhibition of the ssDNA binding activity of Nsp9.

Inhibitor	IC_50_
Myricetin	N.D.
Oridonin	N.D.
Acetone extract of *Sinningia bullata* leaves	N.D.
Water extract of *Nepenthes miranda* leaves	N.D.
Methanol extract of *Nepenthes miranda* leaves	103.4 ± 8.2 μg/mL
Ethanol extract of *Nepenthes miranda* leaves	138.9 ± 12.2 μg/mL
Acetone extract of *Nepenthes miranda* leaves	72.5 ± 6.0 μg/mL
Ethyl acetate extract of *Nepenthes miranda* leaves	307.7 ± 20.4 μg/mL
*n*-Hexane extract of *Nepenthes miranda* leaves	775.4 ± 30.2 μg/mL

Values show the mean standard deviation of at least three independent experiments.

**Table 2:** Molecular docking analysis of Nsp9.

	Affinity (kcal/mol)	Interaction	Residue (distance, Å)
Plumbagin	−5.7	Hydrogen bond	R39 (3.7), T67 (3.1)
		Hydrophobic	F40 (3.7, 3.7), V41 (3.7), F56 (3.5), I65 (3.7), I91(3.8)
Lupenone	−7.1	Hydrophobic	V41 (3.4, 3.3), F56 (4.0, 3.6), I65 (3.3), T67 (3.8)
Palmitic acid	−4.9	Hydrogen bond	S59 (3.1)
		Hydrophobic	F40 (3.7), V41 (3.4, 3.7), F56 (3.6, 3.7, 3.8), I65 (3.9, 3.8), T67 (3.6)
Stigmast-5-en-3-ol	−7.9	Hydrogen bond	R39 (3.7)
		Hydrophobic	V41 (3.9, 3.7), F56 (3.5, 3.4), I65 (3.1), I91 (3.7)
Neophytadiene	−5.1	Hydrophobic	R39 (3.9, 4.0), F40 (3.7), V41 (3.7), F56 (3.8, 3.8, 3.6), T67 (3.8)

## Data Availability

Data are contained within the article.
